# Mathematical Modeling of Growth and Paclitaxel Biosynthesis in *Corylus avellana* Cell Culture Responding to Fungal Elicitors Using Multilayer Perceptron-Genetic Algorithm

**DOI:** 10.3389/fpls.2020.01148

**Published:** 2020-08-11

**Authors:** Mina Salehi, Siamak Farhadi, Ahmad Moieni, Naser Safaie, Hamed Ahmadi

**Affiliations:** ^1^Department of Plant Genetics and Breeding, Faculty of Agriculture, Tarbiat Modares University, Tehran, Iran; ^2^Department of Plant Pathology, Faculty of Agriculture, Tarbiat Modares University, Tehran, Iran; ^3^Bioscience and Agriculture Modeling Research Unit, Department of Poultry Science, Tarbiat Modares University, Tehran, Iran

**Keywords:** secondary metabolite, endophytic fungus, cell extract, culture filtrate, artificial neural network

## Abstract

Paclitaxel is the top-selling anticancer medicine in the world. *In vitro* culture of *Corylus avellana* has been made known as a promising and inexpensive strategy for producing paclitaxel. Fungal elicitors have been named as the most efficient strategy for enhancing the biosynthesis of secondary metabolites in plant cell culture. In this study, endophytic fungal strain HEF_17_ was isolated from *C. avellana* and identified as *Camarosporomyces flavigenus*. *C. avellana* cell suspension culture (CSC) elicited with cell extract (CE) and culture filtrate (CF) derived from strain HEF_17_, either individually or combined treatment, in mid and late log phase was processed for modeling and optimizing growth and paclitaxel biosynthesis regarding CE and CF concentration levels, elicitor adding day, and CSC harvesting time using multilayer perceptron-genetic algorithm (MLP-GA). The results displayed higher accuracy of MLP-GA models (0.89–0.95) than regression models (0.56–0.85). The great accordance between the predicted and observed values of output variables (dry weight, intracellular, extracellular and total yield of paclitaxel, and also extracellular paclitaxel portion) for both training and testing subsets supported the excellent performance of developed MLP-GA models. MLP-GA method presented a promising tool for selecting the optimal conditions for maximum paclitaxel biosynthesis. An Excel^®^ estimator, HCC-paclitaxel, was designed based on MLP-GA model as an easy-to-use tool for predicting paclitaxel biosynthesis in *C. avellana* CSC responding to fungal elicitors.

## Introduction

Paclitaxel is a potent mitotic inhibitor that is utilized for treating breast, lung and ovarian cancers, and Kaposi’s sarcoma (Weaver, 2014), so that it has been entitled the top-selling anticancer medicine in the world (Goodman and Walsh, 2001). Also, this impactful chemotherapeutic agent is used for off-label treatment of endometrial, gastroesophageal, prostate, cervical, and head and neck cancers (Weaver, 2014). Invaluable secondary metabolite “paclitaxel” was initially extracted from *Taxus* bark (Wheeler et al., 1992). But harvesting the bark of these valuable species in the natural areas speedily exceeded levels deemed as a sustainable one, and critical over-harvesting has caused *Taxus* wild populations to be on the brink of extinction worldwide (Shinwari and Qaiser, 2011). Plant cell factories are a promising environmentally sustainable alternative to paclitaxel mass production (Salehi et al., 2017; Espinosa-Leal et al., 2018; Salehi et al., 2019b; Salehi et al., 2019c). The rising demand for paclitaxel and *Taxus* recalcitrant behavior under *in vitro* conditions have caused extensive effort toward finding alternatives for producing this invaluable secondary metabolite.

*In vitro* culture of hazel (*Corylus avellana*, European filbert) has been made known as a promising and inexpensive strategy for producing paclitaxel (Gallego et al., 2017; Salehi et al., 2017; Salehi et al., 2018c; Salehi et al., 2019b; Salehi et al., 2019c; Farhadi et al., 2020; Salehi et al., 2020). Biosynthesizing bioactive compounds in plants is influenced by various factors (Torkamani et al., 2014; Salehi et al., 2017; Salehi et al., 2018a; Salehi et al., 2018b; Salehi et al., 2018c; Salehi et al., 2019a; Salehi et al., 2019b; Salehi et al., 2019c; Salehi et al., 2019d). Previous studies (Salehi et al., 2019b; Salehi et al., 2019c; Farhadi et al., 2020; Salehi et al., 2020) demonstrated the positive influences of cell extract (CE) and culture filtrate (CF) of endophytic fungi on paclitaxel biosynthesis in cell suspension culture (CSC) of *C. avellana*. Fungal elicitor type, concentration and adding time, and also exposure time of cell culture (CSC harvesting time) should be optimized to achieve the maximum biosynthesis of paclitaxel in *C. avellana* CSC (Salehi et al., 2019b; Salehi et al., 2019c; Farhadi et al., 2020; Salehi et al., 2020). Precise analysis of the effects of these factors and their optimal selection would pave the way for the commercialization of bioprocessing *C. avellana* cells toward paclitaxel mass production. Paclitaxel biosynthesis and its elicitation are complex biological processes since they are affected by several factors and their nonlinear interactions. Optimizing these mentioned factors by experimenting is laborious, costly, and time-consuming. The mathematical models can effectively predict the optimized conditions for a multifactorial process (Struik et al., 2005; Gallego et al., 2011) such as paclitaxel biosynthesis.

Artificial intelligence (AI) technology is the algorithm capable of complex and intelligent computing similar to the routine performance of the human brain (Agatonovic-Kustrin and Beresford, 2000). Artificial neural network (ANN) is an AI method discovering complex nonlinear relationships among input (factors) and output (parameters) data (Patnaik, 1999; Plumb et al., 2005). Indeed, ANN is a brain-inspired method that imitates the way that the human brain works (Agatonovic-Kustrin and Beresford, 2000). It processes information and makes decision in systems involving vagueness and uncertainty (Patnaik, 1999; Gago et al., 2010). This technology has been widely used as a predictive instrument in a broad range of fields including ecology, food science, agriculture, environmental sciences, plant biology, pharmaceutical research, and biotechnology (Hilbert and Ostendorf, 2001; Daniel et al., 2008; Huang, 2009; Arab et al., 2018; Hesami et al., 2019a; Hesami et al., 2019b; Hesami et al., 2019c; Sheikhi et al., 2020). Multilayer perceptron (MLP), one of the most popular types of ANN, exhibits superior predictive ability as compared to traditional statistical methods to approximate the mathematical functions for analyzing and interpreting different unforeseeable data sets (Ahmadi and Golian, 2011; Jamshidi et al., 2016). However, training and designing of ANN face several problems. One of the biggest problems is assigning the weights in ANN structure which displays the direct influence on model performance. Basically, the network architecture and learning algorithm parameters control the weights. Also, other network parameters including the number of memory taps, the number of hidden layers and nodes and learning rates could influence ANN performance (Tahmasebi and Hezarkhani, 2009). To overcome these mentioned problems, ANN is hybridized with other optimization methods including genetic algorithm (GA) (Plumb et al., 2005; Shao et al., 2007; Ahmadi and Golian, 2011; Eftekhari et al., 2018; Sheikhi et al., 2020).

**Figure 1:**
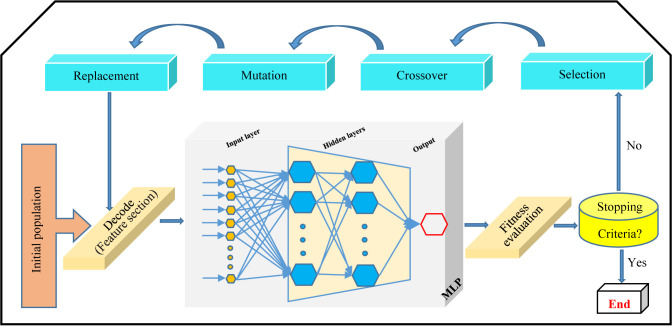
Steps of operation of multilayer perceptron-genetics algorithm (MLP-GA) intelligence.

GA is the evolutionary algorithm making superb solutions to problems and has been applied for bioprocess optimization in plant biology (Osama et al., 2015; Jamshidi et al., 2016; Arab et al., 2018). Indeed, GA is a search algorithm inspired by natural selection and genetics concepts (Holland, 1992). The fundamental principles of GA are the creation of an initial population of search solutions (chromosomes), and then elite search solutions were selected for crossover using a roulette wheel selection method, which will ultimately be the best solution (fittest chromosome) (optimal value) among them (**Figure 1**).

Multilayer perceptron-genetic algorithm (MLP-GA), integrating MLP with GA (**Figure 1**), causes achieving an accurate model for prediction and optimization of biological process (Jamshidi et al., 2016; Arab et al., 2018; Eftekhari et al., 2018).

The objectives of this research were (a) to isolate endophytic fungi from *C. avellana* grown in Iran, (b) to develop regression and MLP-GA models to predict output variables “dry weight (DW), intracellular paclitaxel, extracellular paclitaxel, total yield of paclitaxel and extracellular paclitaxel portion” based on input variables “CE and CF concentration levels, elicitor adding day, and CSC harvesting time”, (c) to compare regression and MLP-GA performance in term of prediction accuracy of output variables, (d) to optimize the mentioned factors for maximum biosynthesis of paclitaxel, (e) to detect the most important factors for maximum biosynthesis of paclitaxel, and (f) to design an Excel^®^ estimator which can easily be applied to predict the total yield of paclitaxel in *C. avellana* CSC based on input variables.

## Materials and Methods

### Isolation of Endophytic Fungi

Healthy samples of the bud, stem, bark, and leaves were obtained from *C. avellana* grown in Iran during June to September 2018. The surfaces of the samples were sterilized as described by Salehi et al. (2018c; 2019b). The surface-sterilized plant samples were cut and transferred on PDAC [potato dextrose agar (PDA); supplemented with 250 mg l^−1^ Chloramphenicol] in unique Petri dishes (100 × 15 mm), incubated at 25 °C. After the growth of endophytic fungi, the pure cultures of the isolates were established by hyphal tip culture (Strobel et al., 1996). All fungal endophytes were numbered as HEF# series and stored at 4 °C.

### Molecular Identification of Endophytic Fungus

Fungal endophyte was cultured in potato dextrose broth (PDB) and incubated in a shaker incubator at 25 °C and 110 rpm for 10 days. The extraction of fungal genomic DNA was done as described by Salehi et al. (2018c; 2019b). The partial sequences of internal transcribed spacer (ITS) fragments (ITS1-5.8S-ITS2) and actin gene (*ACT*) were used to obtain DNA sequence information. ITS fragments were amplified using universal primers ITS1 and ITS4 (White et al., 1990) and *ACT* using primer pair ACT-512F and ACT-783R (Carbone and Kohn, 1999). PCR reaction mixtures (25 µl) consisted of 1 µl genomic DNA (~100 ng), 1 µl forward and reverse primers (10 pM), and 12.5 µl Premix Taq (TaKaRa Biotechnology Ltd., Japan), and 10.5 µl PCR ultrapure water. PCR reaction programs were an initial denaturation at 94 °C for 3 min, followed by 30 cycles of denaturation (94 °C for 30 s), annealing [56 °C (ITS) and 59 °C (ACT) for 30 s], extension (72 °C for 1 min), and a final extension at 72 °C for 5 min. PCR product analysis and purification, sequencing and the phylogenetic analysis were made as described previously (Salehi et al., 2018c; Salehi et al., 2019b).

### Elicitation of *C. avellana* Cell Culture

*C. avellana* CSC was established as described by Salehi et al. (2017; 2018c; 2019b; 2019c). The elicitors (CE and CF) were prepared as described previously (Salehi et al., 2019b). For elicitation, 1.5 ± 0.1 g of *C. avellana* cells (fresh mass) was cultured in 100 ml flasks containing 30 ml MS medium supplemented with 2 mg l^−1^ 2,4-D and 0.2 mg l^−1^ BAP.

Based on our previous studies (Salehi et al., 2019b; Salehi et al., 2019c; Farhadi et al., 2020), three concentrations [2.5, 5, and 10% (v/v)] of fungal elicitors “CE:CF (100:0, 75:25, 50:50, 25:75, 0:100 v/v)” and also mid (day 13) and late (day 17) log phase of *C. avellana* cell cultures were selected for adding fungal elicitors. Control received an equal volume of water (for CE)/PDB (for CF).

### Cell Growth Measurement

Cell growth was determined by the measurement of cell dry weight (DW). Cell biomass was separated from the culture medium by filtration (Whatman No. 1) and washed with distilled water to remove the residual medium, afterward freeze-dried to a constant weight by a vacuum-freeze drier.

### Quantification of Paclitaxel

*C. avellana* cells were separated from the culture medium by a filter paper (Whatman No. 1). Intracellular and extracellular paclitaxel were extracted from the cells and culture broth using a procedure described by Salehi et al. (2017; 2018c; 2019b). Filtering all samples was performed by 0.22 µm cellulose acetate syringe filters before HPLC analysis. Paclitaxel in the samples was analyzed by HPLC (Waters, USA) with a C18 analysis column (Machereye-Nagel EC 250/4.6 Nucleodur). Each sample (20 µl) was injected and detected at 230 nm using a UV detector. The mobile phase was methanol:water (80:20 v/v) at a flow rate of 1.0 ml min^−1^. The quantification of paclitaxel was based on an external standard of genuine paclitaxel (Sigma).

### Experimental Design

The experiment was conducted based on randomized complete block design (RCBD) with factorial arrangement, three factors containing elicitor type with 10 levels [CE:CF (100:0, 75:25, 50:50, 25:75, 0:100 v/v) and water:PDB (100:0, 75:25, 50:50, 25:75, 0:100 v/v)], concentration with three levels [2.5, 5, and 10% (v/v)], elicitor adding day with two levels (days 13 and 17), and three replicates. The cultures were harvested at 2-day intervals after elicitation until the 23^rd^ day.

### Model Development

The data were randomly divided into a training subset (70%) and a testing subset (30%). The training subset was applied to develop multiple linear regression (MLR) and backward regression and also MLP-GA models, and testing subset was applied to test the predictability of developed models (Shao et al., 2006).

### Regression Analysis

Regression analysis is one of well-known predictive modeling methods. The popularity of these models may be assigned to model parameter interpretability and its ease of use. Here, MLR and backward regression models were used to predict DW, intracellular, extracellular and total yield of paclitaxel, and also extracellular paclitaxel portion. Significance level for the independent variables to include in the model was set at 0.05.

To determine which model component is more important during the modeling process, sensitivity analysis was performed on developed regression models using analysis of variance (ANOVA) and absolute t value (|t value|) corresponding to model coefficients. It is noteworthy that a more important model component displays a higher |t value| (Ahmadi and Golian, 2011; Ahmadi and Rodehutscord, 2017).

### Multilayer Perceptron (MLP) Model

Three-layered feed forward back-propagation neural network was used to define the influences of CE and CF concentration levels, elicitor adding day, and CSC harvesting time on DW, paclitaxel biosynthesis (intracellular, extracellular and total), and extracellular paclitaxel portion. Transfer functions for hidden and output layers were hyperbolic tangent sigmoid (tansig) and linear (purelin), respectively.

ANN capability to process the information is determined by its architecture. Evolutionary algorithms are used for searching the optimal architecture design (Yao, 1999).

### Genetic Algorithm (GA)

The high number of hidden neurons leads to prolong the training time and also overfits the data. Too few hidden neurons lead to a low accuracy rate (Matignon, 2005). GA was used (i) to determine optimal MLP architecture design including the optimal numbers of neurons, and (ii) to optimize the values of input variables (CE and CF concentration, elicitor adding day, and CSC harvesting time) in developed MLP-GA models for maximum paclitaxel biosynthesis and its secretion. An initial population of 50, crossover rate of 0.85, generation number of 500 and mutation rate of 0.01 (Haupt and Haupt, 2004; Abramson, 2007) were set to establish fittest MLP structure and optimize input variables for maximum output variables.

The performance of MLP-GA models is determined by root mean square error (RMSE) and coefficient of determination (R^2^) as reported by Ahmadi (2017), as well as mean absolute percentage error (MAPE) [Eq. (1)].

(1) $$MAPE=\;1/n\;\sum_{i=1}^{n}\left|\frac{\left(y_{act}-y_{est}\right)}{\left(y_{act}\right)}\right|\times 100$$

Where “*y_act_*” are the actual values, “*y_est_*” are the predicted values, and “*n*” is the number of data.

### Sensitivity Analysis of the Models

Sensitivity analysis was done on MLP-GA models to determine the importance degree of the factors (CE and CF concentration levels, elicitor adding day, and CSC harvesting time) on the model parameters (DW, paclitaxel biosynthesis, and its secretion). The sensitivity of DW, paclitaxel biosynthesis (intracellular, extracellular, and total yield), and extracellular paclitaxel portion was determined by the criteria including variable sensitivity error (VSE) value displaying the performance (RMSE) of MLP-GA model when that particular input variable is unavailable in the model. Variable sensitivity ratio (VSR) value was calculated as the ratio of VSE and MLP-GA model error (RMSE value) when all input variables are available. Finally, calculated VSR values were rescaled within the range [0, 1]. The input variable with higher VSR was considered as the higher important variable in the model (Ahmadi and Golian, 2011).

The mathematical codes for the development and evaluation of MLR, backward regression, and MLP-GA models were written using MATLAB (Matlab, 2010) software, and the graphs were made by GraphPad Prism 5 (GraphPad Prism 5, 2005) software. “ANNGA_opt” program coded by MATLAB can be downloaded from https://github.com/hahmadima/ANNGA_opt.

## Results

### Identification of Endophytic Fungus

**Figure 2:**
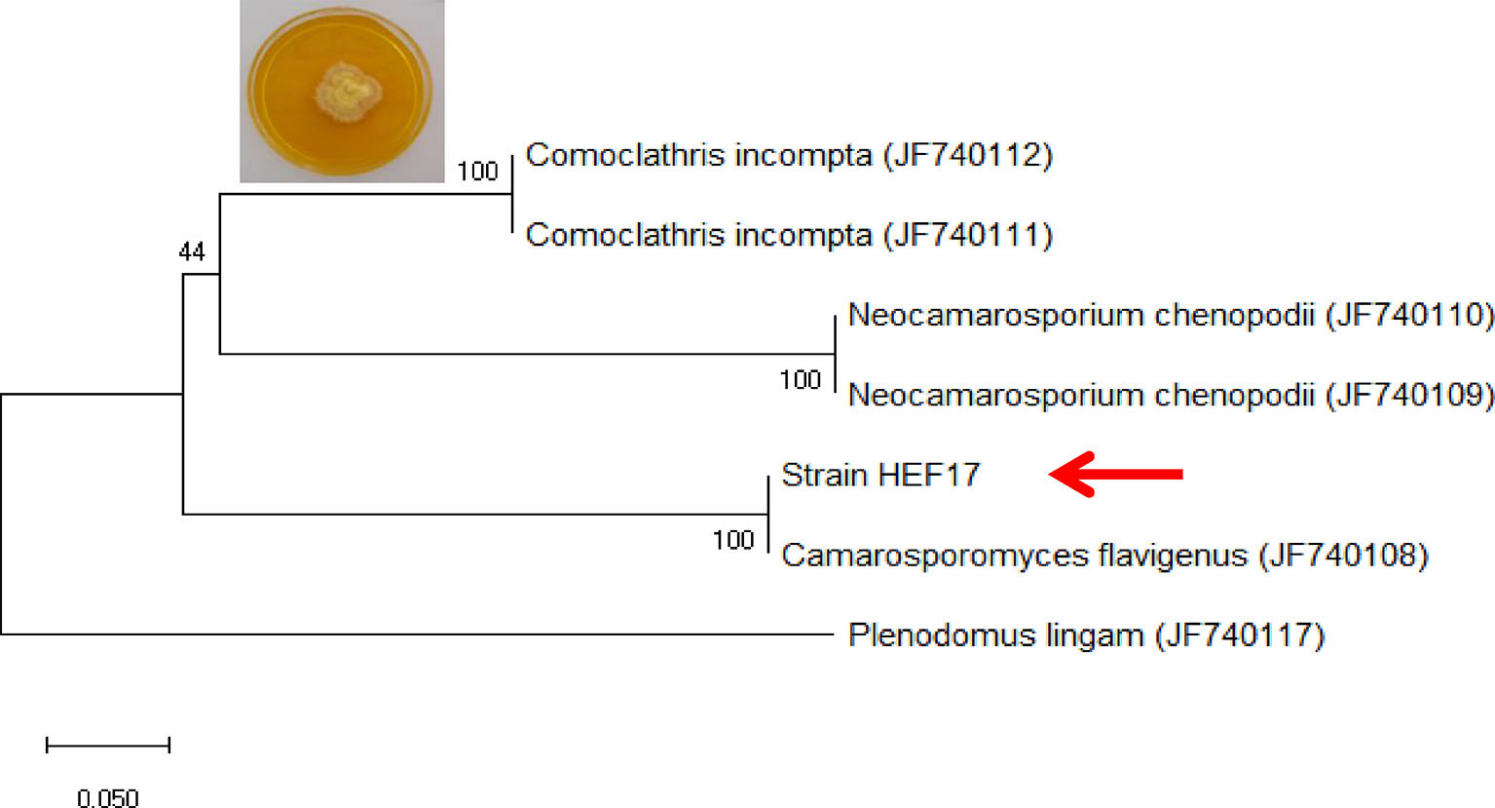
Molecular identification of strain HEF_17_ based on the analysis of the sequences of actin gene. The tree was rooted to *Plenodomus lingam* (CBS 147.24).

Strain HEF_17_ was isolated from the leaf of *C. avellana* and identified as *Camarosporomyces flavigenus* by analysis of the sequences of actin gene (**Figure 2**). Accession numbers used for phylogenetic study were reported by De Gruyter et al. (2013). This is the first report of this endophytic fungus on *C. avellana* tree (matrix nova). The partial sequences of ITS rDNA and *ACT* obtained from *C. flavigenus* strain HEF_17_ were deposited in GenBank (NCBI) under accession numbers MT176168 and MT224136, respectively.

### Regression Analysis

**Figure 3:**
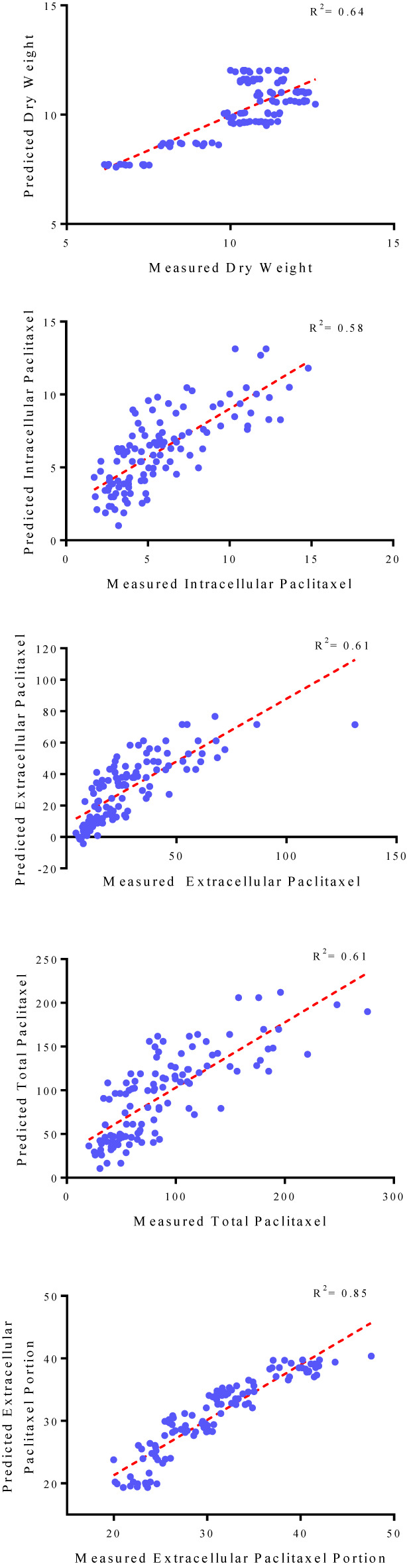
Scatter plot of actual data against predicted values of dry weight, intracellular, extracellular and total yield of paclitaxel, and extracellular paclitaxel portion in *Corylus avellana* cell cultures using backward regression models in the testing subset. The solid line shows fitted simple regression line on scatter points.

Goodness of fit displayed no difference regarding the accuracy of MLR and backward regression for all output variables, 0.66, 0.56, 0.61, 0.58, and 0.85 for DW, intracellular paclitaxel, extracellular paclitaxel, total yield of paclitaxel, and extracellular paclitaxel portion, respectively, for the training subset (**Table 1**). Accordingly, the results of backward regression showed that elicitor adding day and CSC harvesting time are only parameters among the four above-mentioned input variables which influenced DW (**Table 1**). All input variables including CE and CF concentration levels, elicitor adding day and CSC harvesting time are important factors influencing intracellular, extracellular, and total yield of paclitaxel, and also paclitaxel secretion from cells to the culture medium (**Table 1**). R^2^ values for DW, intracellular paclitaxel, extracellular paclitaxel, total yield of paclitaxel, and extracellular paclitaxel portion were estimated 0.64, 0.58, 0.61, 0.61, and 0.85, respectively, for the testing subset (**Figure 3**).

**Table 1:** Backward regression models for estimating growth, paclitaxel biosynthesis, and secretion in *Corylus avellana* cell suspension culture (CSC) treated with fungal elicitors using cell extract (CE) and culture filtrate (CF) concentration levels [% (v/v)], elicitor adding day, and CSC harvesting time (day).

Measured factors	Variable^a^	Coeffcient	Standard error	t value
**Dry weight (g l^-1^)**	Intercept	-0.8950	0.5350	-1.67
	Elicitor adding day	0.0995	0.0325	3.06
	CSC harvesting time	0.4862	0.0239	20.34
R^2^	0.6635			
RMSE	0.9642			
MAPE	4.6650			
**Intracellular paclitaxel (µg g^-1^ DW)**	Intercept	-4.7600	1.3200	-3.60
	CE concentration level	0.4393	0.0531	8.28
	CF concentration level	0.7936	0.0528	15.03
	Elicitor adding day	0.8277	0.0787	10.52
	CSC harvesting time	-0.2170	0.0578	-3.75
R^2^	0.5560			
RMSE	2.3318			
MAPE	59.1400			
**Extracellular paclitaxel (µg l^-1^)**	Intercept	-123.5000	8.9600	-13.78
	CE concentration level	2.6740	0.3600	7.44
	CF concentration level	4.7370	0.3580	13.24
	Elicitor adding day	5.8400	0.5330	10.96
	CSC harvesting time	2.5470	0.3920	6.50
R^2^	0.6060			
RMSE	15.7977			
MAPE	65.60000			
**Total yield of paclitaxel (µg l^-1^)**	Intercept	-242.0000	23.0	-10.51
	CE concentration level	7.1130	0.924	7.70
	CF concentration level	12.7280	0.920	13.84
	Elicitor adding day	15.5000	1.37	11.31
	CSC harvesting time	3.0100	1.01	2.99
R^2^	0.5803			
RMSE	40.5987			
MAPE	67.7200			
**Extracellular** **paclitaxel portion** **(%)**	Intercept	-18.6000	1.4200	-13.07
	CE concentration level	0.1976	0.0571	3.46
	CF concentration level	0.3167	0.0569	5.57
	Elicitor adding day	0.3119	0.0847	3.68
	CSC harvesting time	2.2211	0.0623	35.68
R^2^	0.8549			
RMSE	2.51070			
MAPE	21.18			

a Significant (p ≤ 0.05) variables included in the model. R^2^, coefficient of determination; RMSE, root mean square error; MAPE, mean absolute percentage error.

Goodness of fit of DW model and absolute t values (**Table 1**) showed that out of the investigated input variables, CSC harvesting time (|t value| = 20.3) was the most important parameter affecting DW, followed by elicitor adding day (|t value| = 3.1). Accordingly, CF concentration level (|t value| = 15.0) displayed the highest effect on intracellular paclitaxel, followed by elicitor adding day (|t value| = 10.5), CE concentration level (|t value| = 8.3) and CSC harvesting time (|t value| = 3.8). Also, CF concentration level (|t value| = 13.2) was the most effective component on extracellular paclitaxel, followed by elicitor adding day (|t value| = 11.0), CE concentration level (|t value| = 7.4) and CSC harvesting time (|t value| = 6.5). Furthermore, CF concentration level (|t value| = 13.8) was the most important factor influencing total yield of paclitaxel, followed by elicitor adding day (|t value| = 11.3), CE concentration level (|t value| = 7.7) and CSC harvesting time (|t value| = 3.0). Additionally, CSC harvesting time exhibited the highest effect on extracellular paclitaxel portion (|t value| = 35.7), followed by CF concentration level (|t value| = 5.6), elicitor adding day (|t value| = 3.7) and CE concentration level (|t value| = 3.5) (**Table 1**).

### Multilayer Perceptron-Genetics Algorithm Analysis

**Figure 4:**
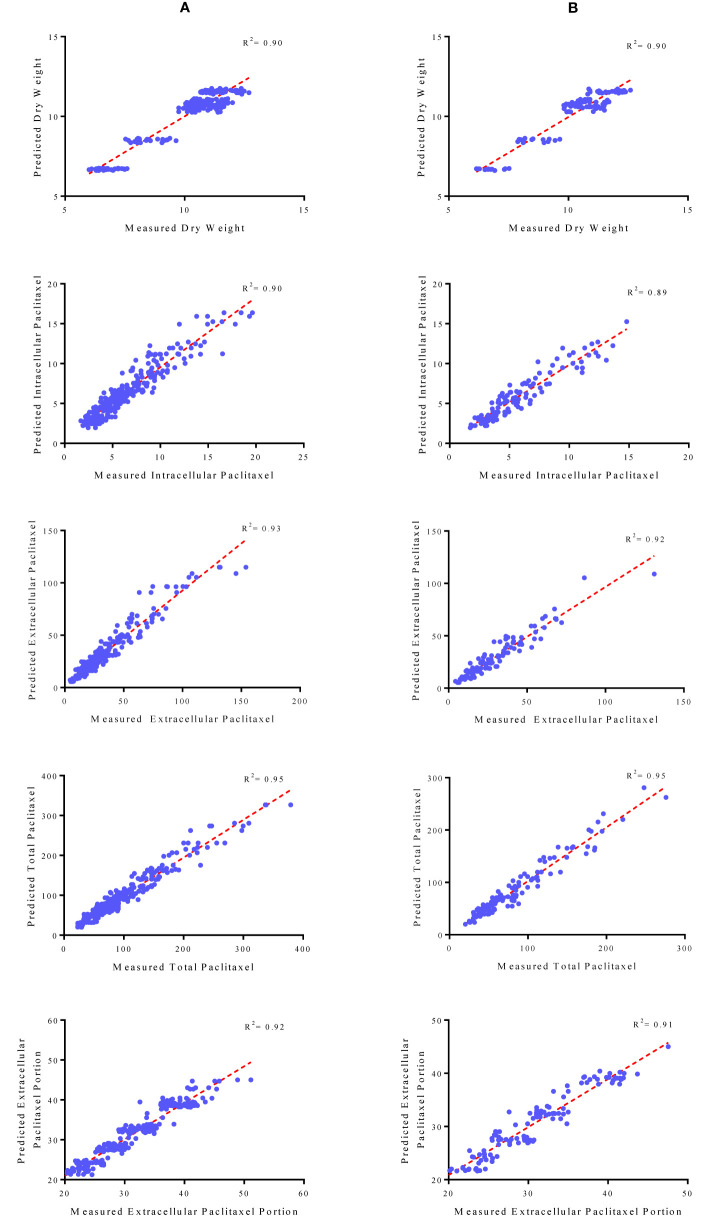
Scatter plot of actual data against predicted values of dry weight, intracellular, extracellular and total yield of paclitaxel and extracellular paclitaxel portion in *Corylus avellana* cell cultures using multilayer perceptron-genetics algorithm (MLP-GA) models in training **(A)** and testing **(B)** subsets. The solid line shows fitted simple regression line on scatter points.

Initially, CE and CF concentration levels, elicitor adding day and CSC harvesting time were used as input variables and DW, intracellular, extracellular and total yield of paclitaxel, and also extracellular paclitaxel portion as output variables. Then, output variables were predicted according to developed MLP-GA models. To evaluate the performance of developed MLP-GA models, the predicted values were plotted against the observed values of training (**Figure 4A**) and testing (**Figure 4B**) subsets. The great accordance between the predicted and observed values of DW, intracellular, extracellular and total yield of paclitaxel, and also extracellular paclitaxel portion was observed for both training and testing subsets (**Figure 4**). Goodness of fit of developed MLP-GA models showed that the developed models could accurately (R^2^ = 0.90, 0.89, 0.92, 0.95, and 0.91) (**Table 2**) predict DW, intracellular, extracellular and total yield of paclitaxel, and also extracellular paclitaxel portion of the testing subset, not used during the training processes (**Figure 4**). Also, developed MLP-GA models displayed the balanced statistical values for both training and testing subsets (**Table 2**).

**Table 2:** Statistics and information on multilayer perceptron-genetics algorithm (MLP-GA) models for growth, paclitaxel biosynthesis and secretion in *Corylus avellana* cell culture.

Measured factors	Neuron number	Training subsets			Testing subsets		
		R^2^	RMSE	MAPE	R^2^	RMSE	MAPE
**Dry weight**	5	0.90	0.53	0.45028	0.90	0.54	0.48036
**Intracellular paclitaxel**	6	0.90	1.14	0.86184	0.89	0.98	0.78311
**Extracellular paclitaxel**	7	0.95	6.78	4.45092	0.92	5.77	4.12229
**Total yield of paclitaxel**	7	0.95	14.27	10.9185	0.95	12.87	9.85688
**Extracellular paclitaxel portion**	5	0.92	1.87	1.54511	0.91	1.89	1.59910

R^2^, coefficient of determination; RMSE, root mean square error; MAPE, mean absolute percentage error.

### Sensitivity Analysis of the Models

To rank the input variables based on their relative importance in the model, VSRs were estimated using all data lines (training and testing subsets). VSRs were obtained for each of output variables (DW, intracellular, extracellular and total yield of paclitaxel, and also extracellular paclitaxel portion) regarding CE and CF concentration levels, elicitor adding day and CSC harvesting time (**Table 3**). Analysis of DW model indicated that DW of *C. avellana* cells was more sensitive to CSC harvesting time (VSR = 0.990), followed by elicitor adding day (VSR = 0.010), CE and CF concentration levels (VSR = 0.004). Intracellular paclitaxel displayed more sensitivity to CE concentration level (VSR = 0.530), followed by CF concentration level (VSR = 0.460), elicitor adding day (VSR = 0.180), and CSC harvesting time (VSR = 0.100). Extracellular paclitaxel showed more sensitivity to CSC harvesting time (VSR = 0.660), followed by CF concentration level (VSR = 0.250), CE concentration level (VSR = 0.110), and elicitor adding day (VSR = 0.100). Accordingly, total yield of paclitaxel exhibited more sensitivity to CE concentration level (VSR = 0.720), followed by CF concentration level (VSR = 0.500), CSC harvesting time (VSR = 0.190), and elicitor adding day (VSR = 0.070). Also, extracellular paclitaxel portion displayed more sensitivity to CSC harvesting time (VSR = 0.810), followed by elicitor adding day (VSR = 0.120), CE concentration level (VSR = 0.080), and CF concentration level (VSR = 0.050) (**Table 3**).

**Table 3:** Importance (according to sensitivity analysis) and optimal levels of the diﬀerent input variables including cell extract (CE), culture filtrate (CF) concentration levels (% (v/v)), elicitor adding day and cell suspension culture (CSC) harvesting time (day) for achieving maximum growth, paclitaxel biosynthesis and secretion in *Corylus avellana* CSC using multilayer perceptron-genetics algorithm (MLP-GA) models.

Criteria	Variable	Importance value (according to VSR^a^)	Optimal level	Output Optimal
**Dry weight (g l^-1^)**	CE concentration level	0.004	5.67	12.04
	CF concentration level	0.004	0.60	
	Elicitor adding day	0.010	15.17	
	CSC harvesting time	0.990	20.78	
**Intracellular paclitaxel (µg g^-1^ DW)**	CE concentration level	0.530	3.37	17.74
	CF concentration level	0.460	5.33	
	Elicitor adding day	0.180	17.00	
	CSC harvesting time	0.100	20.27	
**Extracellular paclitaxel (µg l^-1^)**	CE concentration level	0.110	5.29	124.52
	CF concentration level	0.250	5.75	
	Elicitor adding day	0.100	17.00	
	CSC harvesting time	0.660	20.90	
**Total yield of paclitaxel (µg l^-1^)**	CE concentration level	0.720	3.33	369.67
	CF concentration level	0.500	5.25	
	Elicitor adding day	0.070	17.00	
	CSC harvesting time	0.190	20.95	
**Extracellular paclitaxel portion (%)**	CE concentration level	0.080	4.51	48.07
	CF concentration level	0.050	5.10	
	Elicitor adding day	0.120	17.00	
	CSC harvesting time	0.810	23.00	

a Relative indication of the ratio between the variable sensitivity error and the error of the model when all variables are available. Calculated VSR values were rescaled within range [0, 1].

### Model Optimization

The optimization analysis on developed MLP-GA models was performed using GA to determine the optimal levels of input variables for achieving maximum growth, paclitaxel biosynthesis, and its secretion in *C. avellana* CSC (**Table 3**). The optimization results showed that adding 6.27% (v/v) of 90CE:10CF containing 5.67% (v/v) CE and 0.6% (v/v) CF on 15^th^ day and harvesting CSC 134 h and 38 min after elicitation could result in maximum DW (12.04 g l^−1^) (**Table 3**). The highest content of intracellular paclitaxel (17.74 µg g^−1^ DW) may be produced by adding 8.70% (v/v) of 39CE:61CF containing 3.37% (v/v) CE and 5.33% (v/v) CF on 17^th^ day and harvesting CSC 78 h and 29 min after elicitation (**Table 3**). Also, the results showed that highest extracellular paclitaxel (124.52 µg l^−1^) can be produced by adding 11.13% (v/v) of 48CE:52CF containing 5.29% (v/v) CE and 5.75% (v/v) CF on 17^th^ day and harvesting CSC 93 h and 36 min after elicitation (**Table 3**). Additionally, CSC exposed with 8.58% (v/v) of 39CE:61CF containing 3.33% (v/v) CE and 5.25% (v/v) CF on 17^th^ day and harvesting it 94 h and 48 min after elicitation may obtain the highest total yield of paclitaxel (369.67 µg l^−1^) (**Table 3**). The results of MLP-GA model optimization displayed that adding 9.61% (v/v) of 47CE:53CF containing 4.51% (v/v) CE and 5.10% (v/v) CF on 17^th^ day and harvesting CSC 144 h after elicitation may lead to highest extracellular paclitaxel portion (48.07) (**Table 3**).

### Comparison of MLP-GA and Backward Regression Models

**Figure 5:**
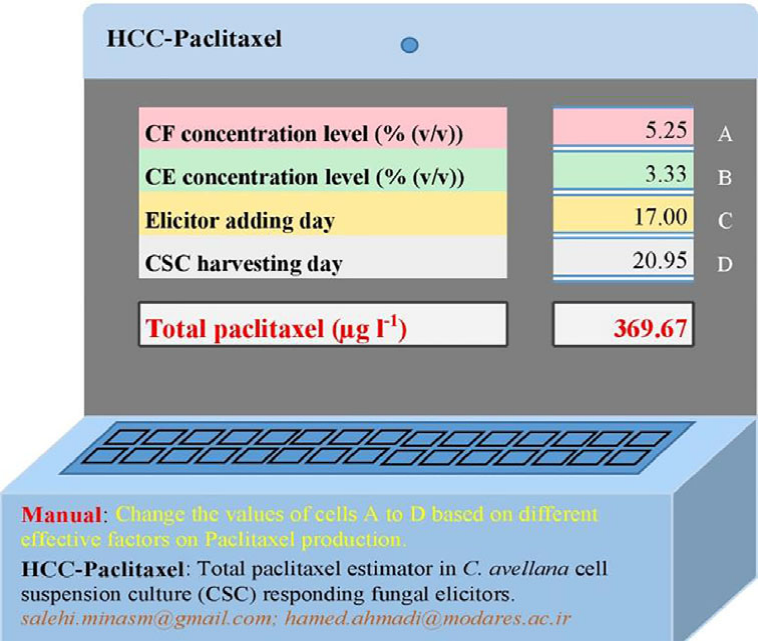
HCC-paclitaxel: an Excel^®^ estimator for predicting total paclitaxel value in *Corylus avellana* cell culture responding fungal elicitors using multilayer perceptron-genetic algorithm (MLP-GA) model. CE, cell extract; CF, culture filtrate. This estimator was presented as Supplementary Material.

The statistical values for MLP-GA models displayed higher prediction accuracy as compared to regression models as estimated R^2^ for MLP-GA *vs.* regression models were: DW = 0.90 *vs.* 0.66, intracellular paclitaxel = 0.90 *vs.* 0.56, extracellular paclitaxel = 0.93 *vs.* 0.61, total yield of paclitaxel = 0.95 *vs.* 0.58, and extracellular paclitaxel portion = 0.92 *vs.* 0.85 (**Tables 1** and **2**). In the end, an Excel^®^ total paclitaxel estimator, namely, HCC-paclitaxel, was created using developed MLP-GA model (**Figure 5**). The mentioned estimator was presented as supplementary material.

## Discussion

Predicting the optimal amount of the effective factors on paclitaxel biosynthesis is highly promising and essential for its production increment and cost decrement. This is the first study on predicting the optimal conditions for maximum paclitaxel biosynthesis in *C. avellana* CSC exposed to fungal elicitors using the mathematical model. To accurately predict the optimal amounts of effective factors (CE and CF concentration levels, elicitor adding day, and CSC harvesting time) on paclitaxel biosynthesis in *C. avellana* CSC, using a trustworthy modeling system is essential.

In this study, regression and MLP-GA modeling were applied to evaluate the relationships among four studied factors “CE and CF concentration levels, elicitor adding day, and CSC harvesting time” and the parameters “DW, intracellular, extracellular, and total yield of paclitaxel and extracellular paclitaxel portion”, and also the possibility of predicting the growth and paclitaxel biosynthesis by the determined factors. Such mathematical predictions have not been described in this area. Higher accuracy of MLP-GA models as compared to regression models (**Tables 1** and **2**) was also reported in previous studies (Jamshidi et al., 2016; Eftekhari et al., 2018).

The fit of regression models was presented by R^2^ (**Figure 3**) for testing subset, suggesting these models can explain 64, 58, 61, 61 and 85% of the variability in DW, intracellular paclitaxel, extracellular paclitaxel, total yield of paclitaxel and paclitaxel extracellular portion, respectively, when they face unseen data.

Our results suggested that MLP-GA models could accurately predict DW, intracellular paclitaxel, extracellular paclitaxel, total yield of paclitaxel and extracellular paclitaxel portion (R^2^ = 0.90, 0.89, 0.92, 0.94, and 0.91, respectively) in the testing subset (**Figure 4**), not used in the training process. Also, the small number of hidden neuron and also closing the errors of training and testing subsets to each other (**Table 2**) suggested that overlearning had not arisen in the training process, and developed MLP-GA models displayed good generalizability when they faced unseen data (Lou and Nakai, 2001; Ahmadi and Golian, 2011). Based on RMSE, R^2^ and MAPE of the training and testing subsets (**Table 2**), it can be concluded that tansig activation function effectively worked for modeling over all experiments. Small RMSE and MAPE (**Table 2**) showed the high potential of MLP-GA models in predicting output variables.

**Figure 6:**
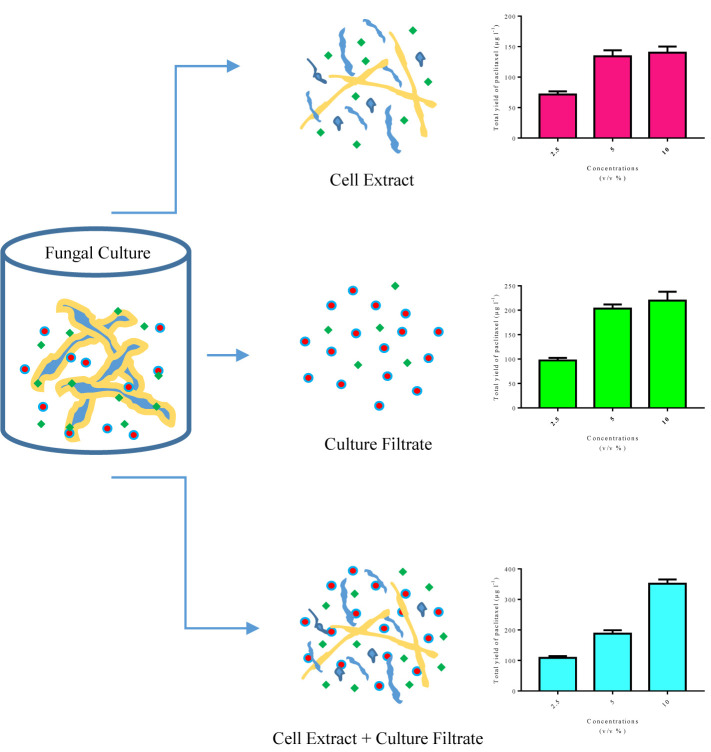
Schematic design of microbe-associated molecular patterns (MAMPs) found in cell extract and culture filtrate, and total yield of paclitaxel in *Corylus avellana* cell cultures exposed with different concentrations of these fungal elicitors derived from *Camarosporomyces flavigenus*.

Regardless of previous studies on the effects of CE and CF concentration levels, elicitor adding day and CSC harvesting time on paclitaxel biosynthesis and secretion, there remains the question to be answered: which input variables are the most important in paclitaxel biosynthesis? As previously mentioned, sensitivity analysis displayed that CE and CF concentration levels are the most important variables affecting total yield of paclitaxel (**Table 3**). Endophytic fungi synthesize microbe-associated molecular patterns (MAMPs). The receptors localized on plant cell surface recognize MAMPs and thus induce plant defense system (Ausubel, 2005). Some of these MAMPs are found only in CE, a number of these exist only in CF, and others are found in both CE and CF with different concentrations (**Figure 6**). Therefore, paclitaxel biosynthesis elicitation potential of these fungal elicitors (CE and CF) is different. Extracellular paclitaxel content is important for paclitaxel production in a continuous system. Sensitivity analysis displayed that CSC harvesting time is the most important factor affecting extracellular paclitaxel (**Table 3**). Paclitaxel biosynthesis is the complex biological process that requires the accurate techniques for modeling and optimization. MLP-GA has been efficiently used to solve problems with extremely difficult and unknown solution in various fields (Jamshidi et al., 2016; Arab et al., 2018; Eftekhari et al., 2018; Sheikhi et al., 2020). A growing interest in ANN has mostly been because of its power in solving the problems in a broad range of fields, their ability for modeling nonlinear and complex relationships, prediction ability of the unseen relationships on the unseen data, and having no need of a specification of data statistical distribution (Mahanta, 2017).

According to the high prediction accuracy of the training and testing subsets, it can be concluded that developed MLP-GA could accurately predict DW, paclitaxel biosynthesis, and secretion in *C. avellana* CSC.

Publishing developed MLP-GA models needs to share the connection weight matrices, which running ANN models requires the especial software. Therefore, we share developed MLP-GA model predicting total paclitaxel with the readers as HCC-paclitaxel Excel^®^ estimator (**Figure 5**).

## Conclusion

This research applied mathematical approaches for modeling and optimizing paclitaxel biosynthesis in *C. avellana* cell culture treated with fungal elicitors for the first time. The great accordance between the predicted and observed values of the output variables (DW, intracellular, extracellular and total yield of paclitaxel, and also extracellular paclitaxel portion) supported the excellent performance of developed MLP-GA models. HCC-paclitaxel Excel^®^ estimator presents an easy-to-use tool to predict total yield of paclitaxel in *C. avellana* cell culture treated with fungal elicitors using MLP-GA model.

## Author Contributions

MS directed the research, carried out all experiments and analyses. AM directed in vitro cell culture and elicitation experiment. NS directed the sections related to fungal elicitation. HA performed data modeling. MS and SF interpreted the results and wrote the manuscript. All authors read and approved the final manuscript.

## Funding

The authors acknowledge Iran National Science Foundation (INSF, No. 97010721), and Research Deputy of Tarbiat Modares University, Tehran for financial support of this research project.

## Conflict of Interest

The authors declare that the research was conducted in the absence of any commercial or financial relationships that could be construed as a potential conflict of interest.
